# Effects of chronic hypoxia on the gene expression profile in the embryonic heart in three Chinese indigenous chicken breeds (*Gallus gallus*)

**DOI:** 10.3389/fvets.2022.942159

**Published:** 2022-08-05

**Authors:** Xiaofeng Li, Abdel-Moneim Eid Abdel-Moneim, Zhongze Hu, Noura M. Mesalam, Bing Yang

**Affiliations:** ^1^College of Animal Science, Anhui Science and Technology University, Fengyang, China; ^2^Biological Applications Department, Nuclear Research Center, Egyptian Atomic Energy Authority, Cairo, Egypt

**Keywords:** signaling pathway, gene, chronic hypoxia, embryonic heart, chicken

## Abstract

Hypoxia exposure (HE) has adverse impacts on the embryonic development of chicken, whereas the mechanism underlying the response of the heart to HE during embryo development in birds is still unclear. Therefore, our study was designed to reveal the hub genes and the signaling pathways linked to chronic hypoxia stress. Thus, the gene expression microarray GSE12675, downloaded from the GEO database, included 12 embryonic heart samples in hypoxia and normoxia of three Chinese indigenous chicken breeds [Shouguang (SG), Tibetan (TB), and Dwarf Recessive White (DRW) chickens]. A total of 653 to 714 breed-specific differentially expressed genes (DEGs) were detected in each pairwise comparison. Gene ontology (GO) showed that the DEGs were mainly involved in biological processes, including vasoconstriction, cell differentiation, and the positive regulation of vasoconstriction. KEGG enrichment revealed that the DEGs were mainly enriched in MAPK, PPAR, insulin, adrenergic signaling in cardiomyocytes, etc. Moreover, 48 genes (e.g., *SGCD, DHRS9, HELQ, MCMDC2*, and *ESCO2*) might contribute to the response of the heart to HE. Taken together, the current study provides important clues for understanding the molecular mechanism of the heart's response to HE during the embryonic period of chicken.

## Introduction

Hypoxia exposure (HE), a frequent natural event during the embryonic stage, has adverse impacts on the development and growth of chicken. HE could cause right ventricular hypertrophy, reduce the body weight, and increase the mortality of chicken embryo, and might be linked to cardiac defect and sudden death syndrome in chicken (1–4). HE altered the developmental trajectories and modified the phenotypes of the developing embryos (2). Also, HE improved the contents of lactic acid, tri-iodothyronine, corticosteroid, and thyroxine in the plasma of chicken during the embryonic stage (3). Villamor et al. reported that HE (15% O_2_) decreased the tolerance of the pulmonary artery to thromboxane A mimetic U-46619, norepinephrine, potassium chloride, endothelin-1, and electrical-field stimulation at embryonic day 19 (4). Inversely, hypoxic hearts showed an increase in left and right ventricular thickness and wall area in chicken embryo (4). In White Leghorn chicken, HE reduced the femoral flow but increased femoral artery resistance by regulating the nitric oxide production at embryonic day 19 (5).

Crossley et al. found that exposure to the three levels of hypoxia (15, 10, and 5% O_2_) depressed the heart rate (HR) and blood pressure (BP) in chicken embryos (6). HE might induce the release of adrenaline and muscarine from autonomic nerve terminals and chromaffin tissue. In embryonic chicken, the tolerance of cardiovascular to hypoxia was involved in the central nervous system and the cholinergic regulation of arterial pressure (6). Severe hypoxia (5% O_2_) resulted in significant HR changes. *In ovo*, the tolerance of HR to severe hypoxia consisted of an initial reduction in HR and the following partial HR recovery. Some factors of the egg, such as catecholamines, might be vital for the survival and response of the chicken embryo to hypoxia (7). HE also reduced the number of cardiomyocytes, restricted growth and heart development, and showed signs of cardiac insufficiency in the chick embryo (8). Hypoxic incubation increased the ratio of the ventricle to body mass on hatching days 11, 15, 17, and 19 and reduced absolute heart mass on hatching day 19. HE affected the proliferation rate and absolute mass of heart at hatching day 15 (8).

Previous studies have shown that a set of genes were involved in the tolerance and adaptation of chicken heart to hypoxia. To enhance the chicken cardiomyocytes in adapting to the hypoxia, the growth factors, stress proteins, and enzymes that were associated with anaerobic glycolysis were observably upregulated. Then, antioxidants and stress proteins were increased in the cardiomyocyte which was reoxygenated after hypoxia (9). HE enhanced the expression of multiple genes including cardiac troponin T, heme oxygenase, and hypoxia upregulated protein 1 in the chicken embryonic heart. Cardiac troponin T was associated with binding tropomyosin to regulate calcium binding and contractility of the heart muscle (10).

It was well-known that Shouguang (SG), Tibetan (TB), and Dwarf Recessive White (DRW) chickens were three Chinese indigenous chicken breeds with many excellent traits, such as outstanding meat quality and excellent environmental adaptability. For example, TB chickens, which inhabited the high-altitude Qinghai-Tibet Plateau for more than 1,000 years, had an adaptive ability to hypoxia (11). In this regard, it is of great significance to reveal potential candidate genes and signaling pathways for those traits. However, the mechanisms of the response to hypoxia in Chinese indigenous chicken breeds during the embryonic period are limited. In the previous study from Li and Zhao (11), the breed-specific differentially expressed gene (DEGs) in the hearts of SG and DRW chickens, and the common DEGs in the heart among the three breeds (SG, TB and DRW chickens) between hypoxia and normoxia have not been analyzed in depth. Therefore, based on the aforementioned research, this study was conducted to clarify the genes and signaling pathways linked to the response of the heart to chronic hypoxia in the three abovementioned chicken breeds.

## Materials and methods

### Sample collection, RNA extraction, and probe hybridization

The current research was performed based on the experiment that was completed by Li and Zhao from China Agricultural University (11). In total, 680 fertilized eggs were obtained from the China Agricultural University's Experimental Chicken Farm. Since the first day of the incubation, 120 SG, 120 DRW, and 100 TB chicken fertilized eggs were kept in the incubator with hypoxia (13% O_2_) or normoxia (21% O_2_) at a temperature of 37.8°C with 60% humidity. At embryonic day 17, the heart tissues from 5 chickens per group were mixed for further pool genome array analysis. Each pool was replicated two times. Subsequently, the total RNA of pool samples was extracted using TRIzol reagent. The hybridized probe array was stained with streptavidin-phycoerythrin in the GeneChip^®^ Fluidics Station 450 (11).

### Differential expression analysis

To explore the DEGs associated with the heart's response in chickens exposed to hypoxia, the data from GSE12675 were analyzed using the GEO2R software (http://www.ncbi.nlm.nih.gov/geo/geo2r). GSE12675, collected from the abovementioned study, included 12 embryonic heart samples in hypoxia and normoxia of three Chinese indigenous chicken breeds (SG, TB, and DRW chickens) with four per breed (11). A *p-*value of < 0.05 with |log_2_Fold Change (FC)| > 1 was identified as the standard for the DEGs.

### Gene ontology and KEGG analysis of the DEGs

To clarify the biological processes closely associated with the DEGs, gene ontology (GO) analysis was conducted using the DAVID database (https://david.ncifcrf.gov/summary.jsp). To annotate the signaling pathways for the DEGs, KOBAS (http://kobas.cbi.pku.edu.cn/kobas3/genelist/) was used for the KEGG analysis of DEGs.

### Reactome analysis and protein classification

Reactome analysis for the DEGs was also implemented with the KOBAS 3.0 software. Moreover, protein classification analysis for the DEGs was tested using the PANTHER classification system (http://pantherdb.org/).

### Protein-protein interaction network

The protein-protein interaction (PPI) network and its further visualization for the DEGs between the two groups were conducted using the STRING database (https://string-db.org/) and the Cytoscape 3.8.0 software (http://www.cytoscape.org/), respectively.

### Hub genes and their functions

The CytoHubba plugin (http://apps.cytoscape.org/apps/cytohubba) was used to obtain the Hub genes. To evaluate the hubs of each gene, the Matthews correlation coefficient (MCC) algorithm was used to calculate the gene connectivity in the PPI network. Then, a new network was created. In the network, the hub nodes were colored according to their importance: from red (the most important) to yellow (the least important) (12). Hub gene functions were summarized with the help of the National Center for Biotechnology Information (NCBI) (https://www.ncbi.nlm.nih.gov/), GeneCards (https://www.genecards.org/), STRING, and previous literature.

## Results

### Overview of the genes in chicken embryonic heart

**Figure 1:**
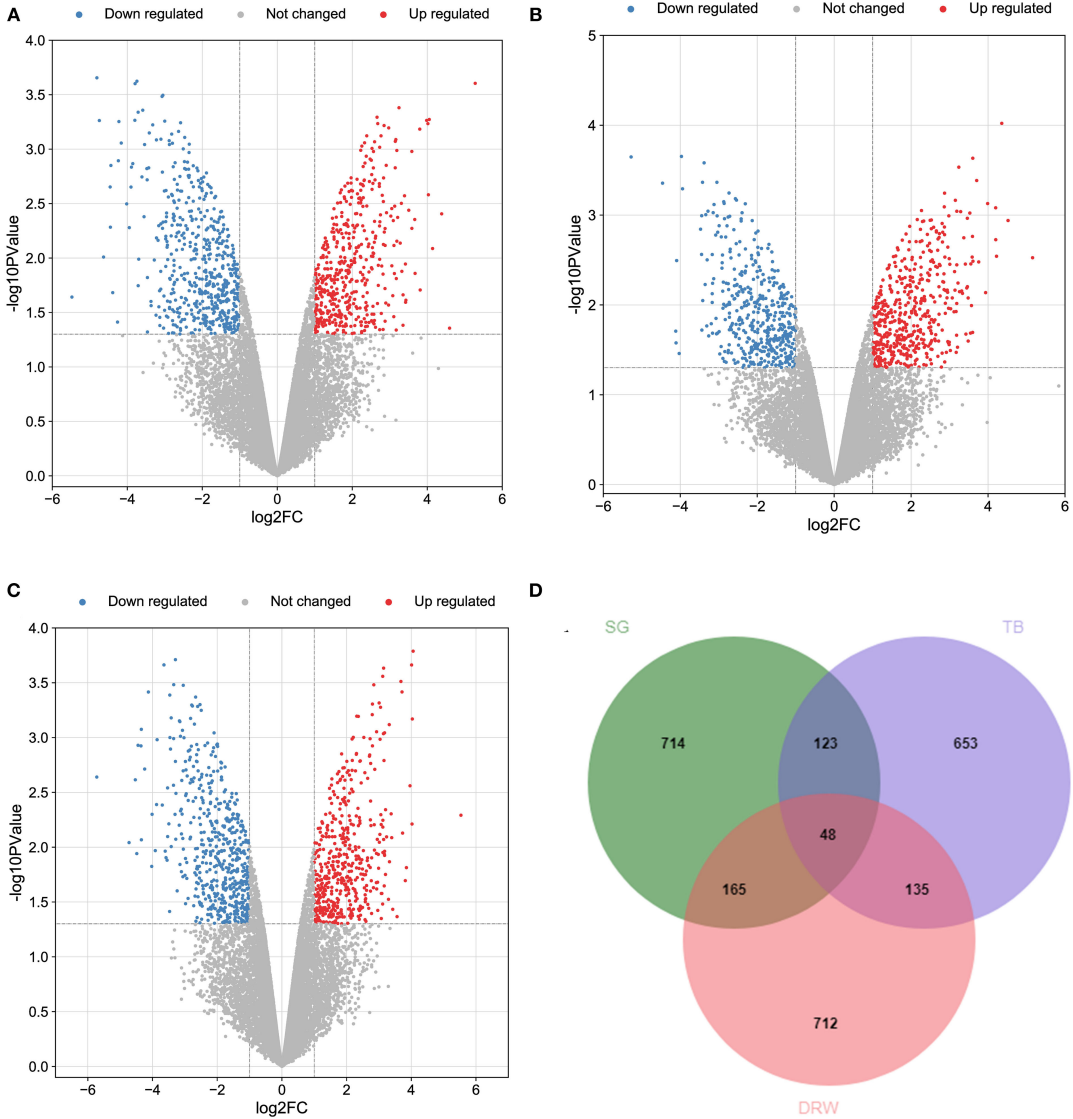
The profile of transcripts and genes in the hearts of chickens treated with hypoxia and normoxia. **(A–C)** revealed the volcano plot of the DEGs in the hearts of SG, TB, and DRW chickens treated with hypoxia and normoxia. The red and blue points represent the upregulated and downregulated genes. The gray spots mean unchanged genes. **(D)** indicated the Venn diagram for the DEGs mentioned above.

Totally 14,086 genes were obtained from the heart samples of the three Chinese indigenous chicken breeds (SG, TB, and DRW chickens). Figures 1A–C represent the volcano plots for the DEGs in SG, TB, and DRW for the comparison of hypoxia vs. normoxia. The Venn diagram for the DEGs identified from the hearts of the three breeds is shown in Figure 1D. The DEGs (Supplementary Files 1–3) of the three breeds' hearts under hypoxia are shown in Supplementary Table 1. A total of 959 to 1,060 DEGs were identified in the three chicken breeds. Compared with the hearts in normoxia, 487, 508, and 491 genes were upregulated, and 463, 451, and 569 genes were downregulated in the SG, TB, and DRW chickens exposed to hypoxia, respectively (Supplementary Table 1).

The breed-specific DEGs in three chicken breeds' hearts under hypoxia are indicated in Supplementary Table 2. Totally 653 to 714 breed-specific DEGs (Supplementary Files 4–6) were identified in the three chicken breeds. Compared with the hearts in the normoxia groups, 330, 347, and 720 genes were upregulated, whereas 384, 306, and 392 genes were downregulated in the hypoxia groups of SG, TB, and DRW chickens, respectively (Supplementary Table 2).

In total, 48 common DEGs in three chicken breeds' hearts between hypoxia and normoxia are displayed in Supplementary Table 3. The common genes included *SGCD, BIRC7, HAVCR1, DHRS9, HELQ, DMRT1, SVOPL, DZANK1, LUZP2, MCMDC2, ESCO2*, and *CSF2*. Most of these genes may be associated with the response of the heart to HE. For example, the protein encoded by *SGCD* is a subcomplex of the dystrophin-glycoprotein complex (DGC). DGC forms a link between the F-actin cytoskeleton and the extracellular matrix. This protein is abundantly expressed in the skeletal and cardiac muscles. *SGCD* mutations are associated with autosomal recessive limb-girdle muscular dystrophy and dilated cardiomyopathy.

Moreover, the top 20 upregulated and downregulated breed-specific DEGs in SG, TB, and DRW chicken hearts under hypoxia are reported in Supplementary Tables 4–9 .

### GO enrichment for the DEGs

**Figure 2:**
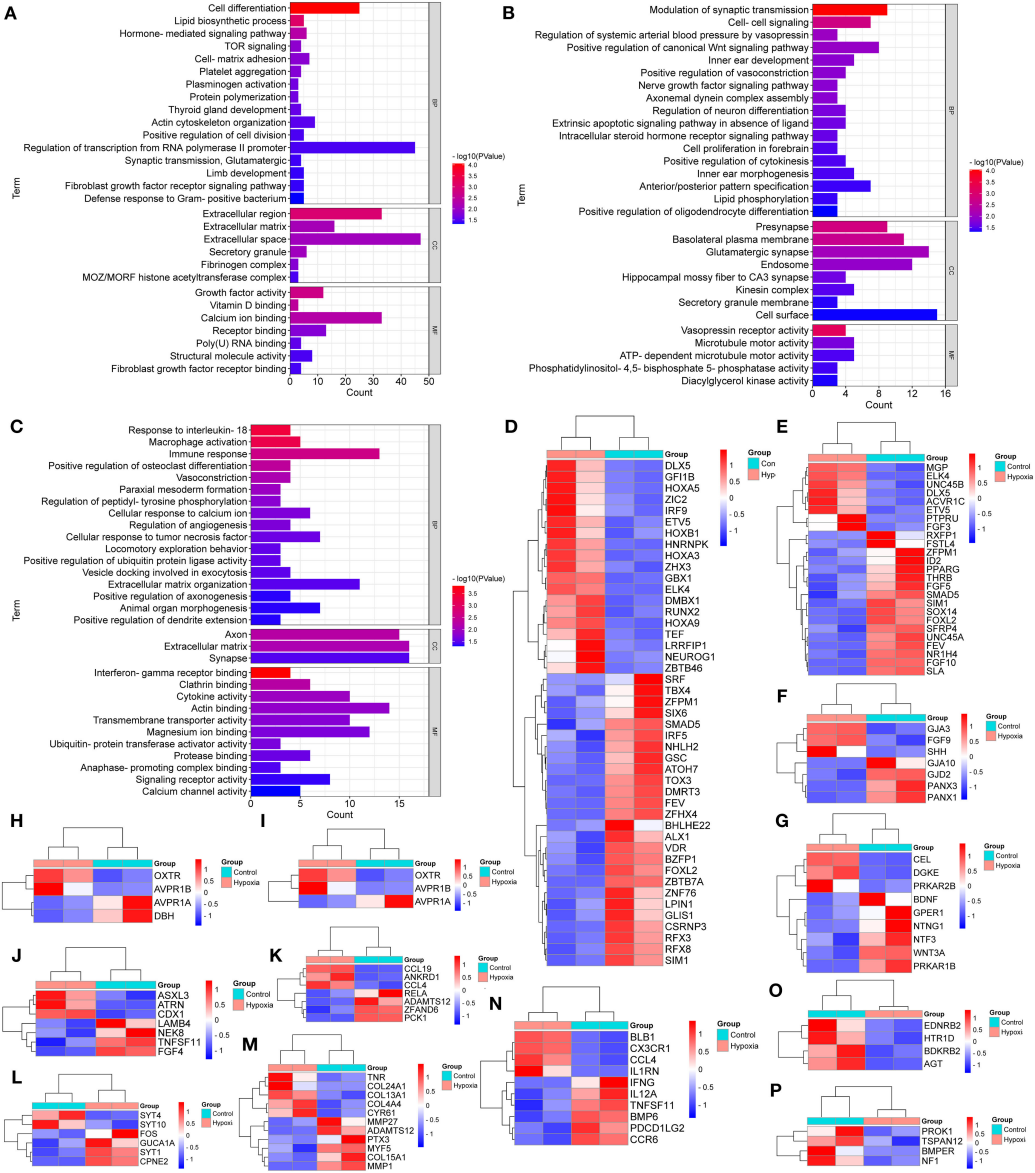
GO Enrichment for the breed-special degs in the hearts of chickens treated with hypoxia and normoxia. **(A–C)** indicated GO enrichment for the breed-special DEGs in the hearts of SG, TB, and DRW chickens treated with hypoxia and normoxia. **(D,E)** indicated the heatmaps of the breed-special DEGs in cell differentiation and the regulation of transcription from RNA polymerase II promoter in SG chicken, respectively. **(F–I)** revealed the heatmaps of the breed-special DEGs in cell-cell signaling; the modulation of synaptic transmission; the positive regulation of vasoconstriction; and the regulation of systemic arterial BP by vasopressin biological processes in TB chicken. **(J–P)** showed the heatmaps of the breed-special DEGs in animal organ morphogenesis, cellular response to tumor necrosis factor, cellular response to calcium ion, extracellular matrix organization, immune response, vasoconstriction, and the regulation of angiogenesis biological processes in DRW chicken.

As shown in Figure 2A, the special DEGs in SG chicken heart were involved in many biological processes, including cell differentiation, lipid biosynthetic process, TOR signaling, cell-matrix adhesion, platelet aggregation, protein polymerization, plasminogen activation, actin cytoskeleton organization, the positive regulation of cell division, synaptic transmission, glutamatergic, and the defense response to Gram-positive bacterium.

Also, as indicated in Figure 2B, the special DEGs in TB chicken heart were involved in various biological processes, such as the positive regulation of vasoconstriction, cell-cell signaling, modulation of synaptic transmission, nerve growth factor signaling pathway, regulation of neuron differentiation, cell proliferation in the forebrain, positive regulation of cytokinesis, anterior/posterior pattern specification, and lipid phosphorylation.

In addition, as revealed in Figure 2C, the special DEGs in DRW chicken heart may play a key role in various biological processes, such as vasoconstriction, animal organ morphogenesis, the response to interleukin-18, macrophage activation, immune response, cellular response to calcium ion, the regulation of angiogenesis, cellular response to tumor necrosis factor, and extracellular matrix organization.

Furthermore, the breed-special DEGs in the regulation of transcription from RNA polymerase II promoter and cell differentiation biological processes in SG chicken are indicated in Figures 2D,E, respectively. The breed-special DEGs in cell-cell signaling, modulation of synaptic transmission, positive regulation of vasoconstriction, and regulation of systemic arterial BP by vasopressin biological processes in TB chicken are shown in Figures 2F–I, respectively; The breed-special DEGs in animal organ morphogenesis, cellular response to tumor necrosis factor, cellular response to calcium ion, extracellular matrix organization, immune response, vasoconstriction, and the regulation of angiogenesis biological processes in DRW chicken are revealed in Figures 2J–P, respectively.

### KEGG enrichment for the DEGs

**Figure 3:**
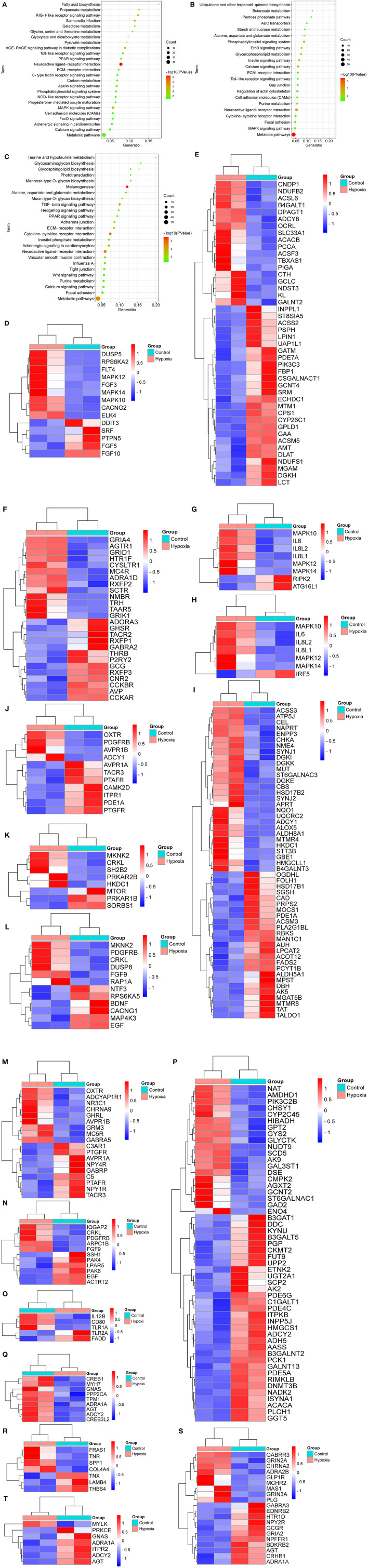
KEGG analysis for the breed-special DEGs. **(A–C)** indicated the KEGG analysis for the breed-special DEGs in the hearts of SG, TB, and DRW chickens treated with hypoxia and normoxia. **(D–H)** showed the heatmaps of the breed-special DEGs in metabolic pathways, MAPK, NLRI, NOD-like receptor, and toll-like receptor signaling pathways in SG chickens. **(I–O)** revealed the heatmaps of the breed-special DEGs in metabolic pathways; calcium, insulin, MAPK; NLRI; regulation of actin cytoskeleton; and toll-like receptor signaling pathways in TB chicken. **(P–T)** showed the heatmaps of the breed-special DEGs in metabolic pathways; adrenergic signaling in cardiomyocytes, ERI, NLRI, and vascular smooth muscle contraction signaling pathways in DRW chicken.

As shown in Figure 3A, the breed-special DEGs in SG chicken heart were mainly involved in adrenergic signaling in cardiomyocytes, PPAR, MAPK, FoxO, calcium, apelin, toll-like receptors, neuroactive ligand-receptor interaction (NLRI), ECM-receptor interaction (ERI), RIG-I-like receptor, AGE-RAGE, fatty acid biosynthesis, metabolic pathways, carbon metabolism, glyoxylate and dicarboxylate metabolism, and progesterone-mediated oocyte maturation.

Also, the breed-special DEGs in TB chicken heart were mainly involved in calcium, ErbB, MAPK, insulin, metabolic pathways, ABC transporters, Gap junction, cell adhesion molecules, glycerophospholipid metabolism, cytokine-cytokine receptor interaction, alanine, aspartate and glutamate metabolism, and toll-like receptor signaling pathways (Figure 3B).

Moreover, the breed-special DEGs in DRW chicken heart were primarily enriched in adrenergic signaling in cardiomyocytes, vascular smooth muscle contraction, ERI, calcium, hedgehog, PPAR, melanogenesis, metabolic pathways, TGF-beta, tight junction, adherens junction, glycosphingolipid biosynthesis, glycosphingolipid biosynthesis, purine metabolism, and Wnt signaling pathways (Figure 3C).

Furthermore, the heatmaps for the breed-special DEGs in metabolic pathways, MAPK, NLRI, NOD-like receptor, and toll-like receptor signaling pathways in SG chicken are shown in Figures 3D–H, respectively; The heatmaps for the breed-special DEGs in metabolic pathways, calcium, insulin, MAPK, NLRI, regulation of actin cytoskeleton, and toll-like receptor signaling pathways in TB chicken are shown in Figures 3I–O, respectively; The heatmaps for the breed-special DEGs in metabolic pathways, adrenergic signaling in cardiomyocytes, ERI, NLRI, and vascular smooth muscle contraction signaling pathways in DRW chicken are revealed in Figures 3P–T, respectively.

### Reactome analysis and protein classification for the DEGs

**Figure 4:**
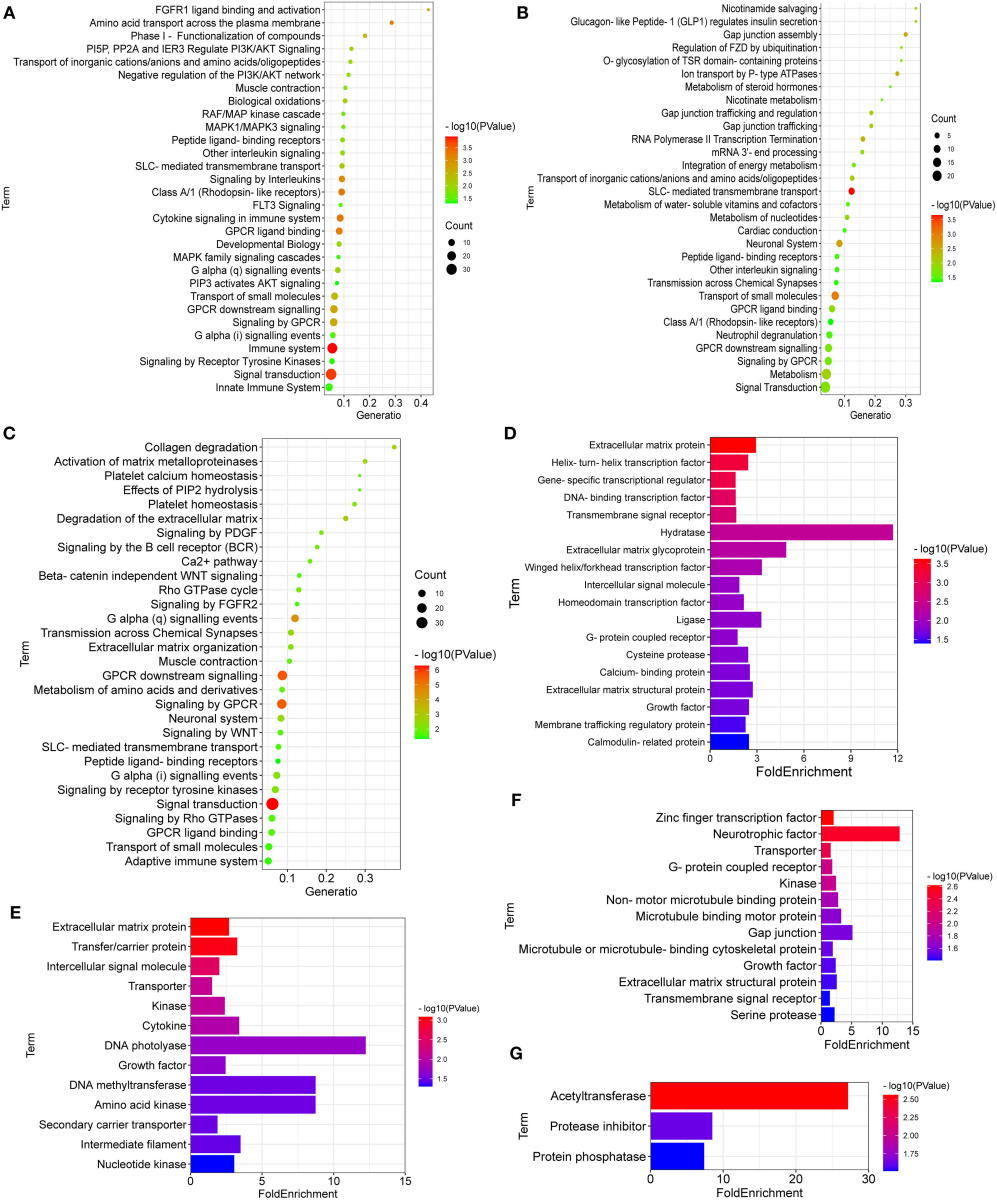
Reactome enrichment and protein class for the breed-special DEGs. **(A–F)** showed Reactome enrichment and protein class for the breed-special DEGs in the hearts of SG, TB, and DRW chickens. **(G)** showed protein class of the common DEGs among the three chicken breeds.

As shown in Figure 4A, the breed-special DEGs in SG chicken heart were mainly enriched in myogenesis, striated muscle contraction; muscle contraction, signal transduction, small molecules transport, signaling by receptor tyrosine kinases, biological oxidations, PI3K/AKT signaling, RAF/MAP kinase cascade, MAPK1/MAPK3 signaling, MAPK family signaling cascades, and PIP3 activates AKT signaling.

In addition, the breed-special DEGs in TB chicken hearts might play an important role in signal transduction; metabolism; SLC-mediated transmembrane transport; neuronal system; GPCR ligand binding; neutrophil degranulation; nucleotides metabolism; transmission across chemical synapses; and Gap junction assembly (Figure 4B).

Moreover, the breed-special DEGs in DRW chicken hearts were mainly involved in striated muscle contraction; signal transduction; muscle contraction; GPCR downstream signaling; G alpha signaling events; adaptive immune system; neuronal system; extracellular matrix organization; Rho GTPase cycle; collagen degradation; activation of matrix metalloproteinases; and platelet homeostasis (Figure 4C).

The DEGs performed protein classification using PANTHER (Figures 4D–G). As shown in Figure 4D, the breed-special DEGs in SG chicken hearts were mainly involved in hydratase, ligase, cysteine protease, extracellular matrix glycoprotein, calmodulin-related protein, helix-turn-helix transcription factor, and membrane trafficking regulatory protein.

The breed-special DEGs in TB chicken might have vital roles in the transporter, kinase, neurotrophic factor, gap junction, microtubule binding motor protein, non-motor microtubule-binding protein; growth factor, zinc finger transcription factor, microtubule or microtubule-binding cytoskeletal protein, and transmembrane signal receptor (Figure 4E).

The breed-special DEGs in DRW chicken might be involved in the transporter, kinase, DNA photolyase, growth factor, DNA methyltransferase, intermediate filament, cytokine, transfer/carrier protein, nucleotide kinase, amino acid kinase, extracellular matrix protein, intercellular signal molecule, and secondary carrier transporter (Figure 4F). Of particular note was that the common DEGs among three chicken breeds might play a vital role in acetyltransferase, protease inhibitor, and protein phosphatase (Figure 4G).

### PPI network and hub genes and their functions

**Figure 5:**
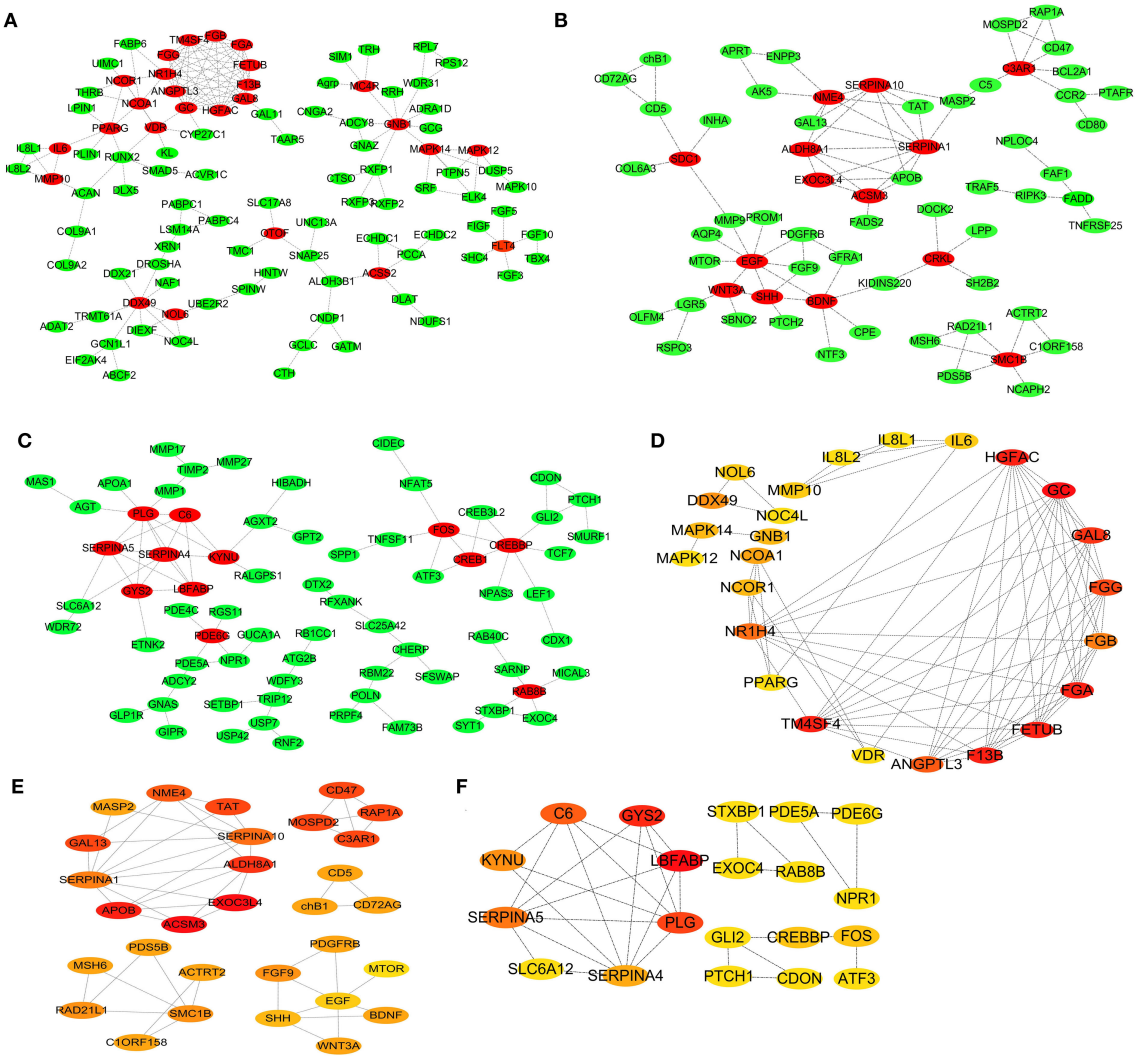
PPI Network and hub genes for the breed-special DEGs. **(A–F)** represented the PPI network and the Hub genes analysis for the breed-special DEGs in the hearts of SG, TB, and DRW chickens. The significance of genes highlighted in red, orange, and yellow was gradually decreased in the response of the hearts exposed to hypoxia.

To further reveal the core genes, the DEGs in the three chicken breeds, namely SG, TB, and DRW, were implemented in the analysis of the PPI networks, and the results are shown in Figures 5A–C, respectively. As shown in Figure 5A, many genes such as *FETUB, HGFAC, GAL8, FGG, GC*, and *DDX49* might be related to the response of SG chicken heart to chronic hypoxia. Similarly, *ALDH8A1, C3AR1, ACSM3, EXOC3L4, TAT, RAP1A, APOB*, and *GAL13* might be closely associated with the response of TB chicken heart to chronic hypoxia (Figure 5B). Many genes, including *LBFABP, STXBP1, SLC6A12, NPR1, CDON, GYS2, PLG, C6, SERPINA5, KYNU*, and *PDE5A* might be closely linked to the response of DRW chicken heart to chronic hypoxia (Figure 5C).

As shown in Figure 5D, the top 25 hub genes from the DEGs in SG chicken included *GC, F13B, FGA, TM4SF4, FETUB, HGFAC, GAL8, FGG*, and *ANGPTL3*. The top 20 hub genes from the DEGs in TB chicken included *ACSM3, EXOC3L4, APOB, ALDH8A1, C3AR1, TAT, RAP1A, GAL13*, and *MOSPD2* (Figure 5E). The top 20 hub genes from DEGs in DRW chicken included *LBFABP, GYS2, PLG, C6, SERPINA5, KYNU, SERPINA4, FOS*, and *CREBBP* (Figure 5F).

## Discussion

### Genes related to the response of chicken heart to chronic hypoxia

In this study, we found 48 common DEGs in the heart of three chicken breeds between hypoxia and normoxia, including *SGCD, DHRS9, HELQ, MCMDC2*, and *ESCO2*. That is to say, those genes are likely to be closely associated with the response of the chicken heart to chronic hypoxia. For example, our data showed that the expression of *SGCD* was significantly downregulated in the hearts of SG (decreased by 89%) and DRW (decreased by 66%) chickens, but upregulated in TB (increased by 230%) chickens under hypoxia, which was consistent with the report that *SGCD* might be involved in chicken cardiomyopathy and muscular dystrophy. *SGCD* gene knockout could result in progressive heart muscle dysfunction in drosophila (13). Besides, TGF beta activation and SMAD signaling played a vital role in cardiac muscle function and injury in *SGCD* null flies (13).

*DHRS9*, named dehydrogenase/reductase 9, is a powerful biomarker for human regulatory macrophages. *DHRS9* expression distinguished Mregs from a set of antigen-presenting cells, such as DC-10 and PGE2-induced myeloid-derived suppressor cells (14). In our study, the expression of *DHRS9* in the hearts of SG, TB, and DRW chickens in hypoxia was significantly higher than that in normoxia, which agrees with the report that *DHRS9* was obviously upregulated in patients with the cardiorenal syndrome, and it might serve as a potential biomarker for predicting the cardiorenal syndrome (15). Deniz et al. analyzed the gene expression profiles of patients with degenerative mitral regurgitation (DMR) in sinus rhythm and atrial fibrillation and found that *DHRS9* might have a structural remodeling role in the extracellular matrix and cellular stress response (16).

*HELQ*, a DNA helicase, played an important role in DNA lesions repair. The antitumor activities of *HELQ* might be associated with upregulated expression of the DNA damage-related proteins CHK1 and RAD51 (17). During the homologous recombination of cells, *HELQ* deficiency compromised the end-joining and single-strand annealing pathways and resulted in the bias toward the long-tract gene conversion tracts (18).

MCMDC2, named minichromosome maintenance domain containing 2, might play an important role in the formation and stabilization of DNA strands. During meiotic recombination, MCMDC2 promoted homolog alignment and provided the basis for inter-homolog crossover formation (18). MCMDC2 played a crucial function in meiotic recombination (19). MCMDC2 was vital for homologous sequences invasion by stabilization of recombination intermediates following strand invasion, both of which were needed to drive stable DSB repair *via* recombination (20). In this study, the expression of *MCMDC2* gene in the hearts of SG, TB, and DRW chickens under hypoxia was obviously higher than those under normoxia, hinting that *MCMDC2* might enhance homolog alignment and DNA strand stabilization in chicken heart development under hypoxia during the embryonic stage.

ESCO2, an acetyltransferase, is required for neuronal differentiation and sister chromatid cohesion. *ESCO2* overexpression enhanced the differentiation of neural progenitor cells and P19 embryonic carcinoma cells. On the contrary, the *ESCO2* knockdown blocked the differentiation of the above-mentioned cells. Importantly, Notch protein mediated the *ESCO2* effects (21). The *ESCO1*-dependent modification of SMC3 regulated the cohesin activities, such as transcriptional control, DNA repair, chromosome loop stabilization, and formation (21). *ESCO2* was upregulated during fin regeneration and specifically within the blastema. *ESCO2* knockdown significantly reduced the *CX43*/*GJA1* expression, which was required for cell-cell communication (22).

### Signaling pathways linked to the response of chicken heart to chronic hypoxia

The response of the chicken heart to chronic hypoxia was mainly associated with multiple signaling pathways, including MAPK, PPAR, insulin, metabolic pathways, ERI, adrenergic signaling in cardiomyocytes, and vascular smooth muscle contraction. The MAPK signaling pathway was likely to participate in cardiac function regulation under hypoxia. Research showed that the MAPK signaling pathway mediated the hypoxia/reoxygenation (H/R)-induced injury in cardiomyocytes (23). The key proteins in MAPK/JNK signaling pathway, which were inhibited by the miR-155 inhibitor, were significantly upregulated in the H/R cardiomyocytes, and BAG5 overexpression enhanced the protective effect of those proteins on the cell injury induced by H/R. Moreover, the *HIF1a* expression patterns were altered following different treatments (24). The MAPK signaling pathway also had a vital role in regulating DNMT1/HMGB1-mediated cardiac progenitor cell apoptosis during the hypoxia process (25).

*PPAR*, a vital regulator for lipid metabolism, was linked to maintaining the homeostasis of myocardial energy metabolism, cardiac function, and tissue structure (26–30). Hypoxia increased myocardial lipid accumulation and mitochondrial dysfunction. Hypoxia downregulated the myocardial lipid metabolism-related genes, including *PPAR*α (26). WY14643, a kind of *PPAR*α activator, reduced the lipid accumulation in the myocardium induced by hypoxia, enhanced the left ventricular systolic and mitochondrial functions, and upregulated the *PPAR*α, *PPARGC1A*, and *CPT1A* genes, and downregulated *ACC2* (26). In our study, the PPAR signaling pathway might mediate chicken cardiac response to hypoxia, which was consistent with the report that *PPAR*α also played a vital role in regulating cardiac metabolic remodeling in response to both hypoxia and supplementation of nitrate in diet (27). Metabolism intervention might offer new approaches to the treatment of heart failure (29). *PPAR*γ also powerfully modulated the signaling disorders in the heart and pulmonary vascular wall (30). Chronic hypoxia could result in cardiomyocyte hypertrophy, right ventricle hypertrophy (RVH), and right ventricle systolic pressure. Pioglitazone, a kind of *PPAR*γ agonist, relieved the RVH, pulmonary hypertension, and cardiomyocyte hypertrophy induced by chronic hypoxia (30).

Insulin can eliminate the adverse effects of hypoxia and improve cardiomyocyte viability. Hypoxia reduced the cardiomyocyte viability, increased the autophagy and apoptosis and endoplasmic reticular (ER) stress pathway-associated apoptotic responses accompanied by an increase of pro-apoptotic transcriptional factor, and apoptosis in myocardial cells. In this study, the insulin signaling pathway might have a vital role in chicken cardiac response to hypoxia, which was similar to the report by Liu et al. who found that insulin could effectively relieve autophagy and ER stress and prevent hypoxia-induced cellular apoptosis *via* PI3K/Akt signaling pathway (31). An unusual HR, blood glucose, and mean arterial BP induced by hypoxia could be restored to the normoxia state by an acute insulin supplement (32).

ECM-receptor interaction (ERI) may be related to the response of the chicken heart to hypoxia. The ERI pathway regulated cell proliferation, apoptosis, growth, and differentiation. The dysregulation of the pathway was responsible for cell death (33). Qi et al. reported that yak was well-adapted to the hypoxia and high altitude. To clarify the underlying mechanism behind the adaptation, they conducted a transcriptomic experiment for yaks and found that the heart was the key organ showing adaptive transcriptional changes. Multiple cell proliferation and survival-associated signaling pathways, including ERI, PI3K-Akt, HIF-1, and focal adhesion, might be involved in the adaptation and response of the heart to hypoxia (34). The study from San et al. identified the DEGs in crureus and pectorales between the Arbor Acres and the Zhuanghe dagu chickens and indicated that the ERI pathway was co-enriched in both the tissues. In addition, ERI could regulate the metabolism of intramuscular adipocytes (35).

Adrenergic signaling in cardiomyocytes is very important for maintaining cardiac physiological function and preventing cardiac diseases. Beta-adrenergic stimulation highly restricted the cAMP distribution and promoted the phosphorylation of proteins for the contractile responses of cardiomyocytes. Moreover, the interaction between beta-adrenergic signaling and other receptor-stimulated signaling cascades changed the beta-adrenergic signaling for proper contractility in the myocardium (36). Beta-adrenergic stimulation rapidly increased G alpha palmitoylation in cardiomyocytes. This palmitoylation kinetics was temporally consistent with the downstream production of cAMP and contractile responses. The plasma membrane-localized palmitoyl acyltransferase DHHC5 is an important mediator of the stimulus-dependent palmitoylation in cardiomyocytes (37).

However, there are two deficiencies in the present study: (1) the lack of verification for the transcriptome sequencing results, and (2) the lack of further studies on the mechanisms of the important genes and signaling pathways potentially relevant to the chronic hypoxia-induced chicken embryonic heart.

## Conclusion

In the study, we found diverse signaling pathways (including MAPK, PPAR, insulin, ERI, and adrenergic signaling pathways) and many genes, such as *SGCD, DHRS9, HELQ, MCMDC2*, and *ESCO2* might contribute to the response of the chicken heart to HE. This study provided a valuable clue for the in-depth understanding of the molecular mechanism of the adaptability of the chicken heart to chronic hypoxia.

## Data availability statement

The datasets presented in this study can be found in online repositories. The names of the repository/repositories and accession number(s) can be found in the article/Supplementary material.

## Ethics statement

The animal study was reviewed and approved by the Institutional Animal Care and Use Committee (ECASTU-2015-P08) of Anhui Science and Technology University, China.

## Author contributions

XL analyzed and visualized the results and wrote the manuscript. BY and ZH conceived the study. NM and A-MA-M revised the scientific English. All authors reviewed the manuscript.

## Funding

This study was supported by the Natural Science Foundation of Anhui Provincial Education Department (No. KJ2020A0083) and the Talent Introduction Program of Anhui Science and Technology University (No. DKYJ202003).

## Conflict of interest

The authors declare that the research was conducted in the absence of any commercial or financial relationships that could be construed as a potential conflict of interest.

## Publisher's note

All claims expressed in this article are solely those of the authors and do not necessarily represent those of their affiliated organizations, or those of the publisher, the editors and the reviewers. Any product that may be evaluated in this article, or claim that may be made by its manufacturer, is not guaranteed or endorsed by the publisher.

## References

[1] Azzam MA Mortola JP. Organ growth in chicken embryos during hypoxia: implications on organ “sparing” and “catch-up growth”. Respir Physiol Neurobiol. (2007) 159:155–62. 10.1016/j.resp.2007.06.003 17652035

[2] Dzialowski EM von Plettenberg D Elmonoufy NA Burggren WW. Chronic hypoxia alters the physiological and morphological trajectories of developing chicken embryos. Comp Biochem Physiol A Mol Integr Physiol. (2002) 131:713–24. 10.1016/S1095-6433(02)00009-0 11897182

[3] Hassanzadeh M Bozorgmehri Fard MH Buyse J Bruggeman V Decuypere E. Effect of chronic hypoxia during embryonic development on physiological functioning and on hatching and post-hatching parameters related to ascites syndrome in broiler chickens. Avian Pathol. (2004) 33:558–64. 10.1080/03079450400013188 15763722

[4] Villamor E Kessels CGA Ruijtenbeek K van Suylen RJ Belik J De Mey JGR. Chronic in ovo hypoxia decreases pulmonary arterial contractile reactivity and induces biventricular cardiac enlargement in the chicken embryo. Am J Physiol Regul Integr Comp Physiol. (2004) 287:R642–51. 10.1152/ajpregu.00611.2003 15117730

[5] Iversen NK Wang T Baatrup E Crossley DA. The role of nitric oxide in the cardiovascular response to chronic and acute hypoxia in White Leghorn chicken (*Gallus domesticus*). Acta Physiol. (2014) 211:346–57. 10.1111/apha.12286 24673734

[6] Crossley DA Burggren WW Altimiras J. Cardiovascular regulation during hypoxia in embryos of the domestic chicken *Gallus gallus*. Am *J Physiol Regul Integr Comp Physiol*. (2003) 284:R219–26. 10.1152/ajpregu.00654.2001 12388452

[7] Nechaeva M Alekseeva T Dobretsov M Kubasov I. Chicken embryos can maintain heart rate during hypoxia on day 4 of incubation. J Comp Physiol B. (2020) 190:361–70. 10.1007/s00360-020-01274-5 32198537

[8] Safaei P Khadjeh G Tabandeh MR Asasi K. Role of cardiac hypoxia in the pathogenesis of sudden death syndrome in broiler chickens-A metabolic and molecular study. Acta Vet Hung. (2021) 69:43–9. 10.1556/004.2021.00004 33764895

[9] Piacentini L Karliner JS. Altered gene expression during hypoxia and reoxygenation of the heart. Pharmacol Ther. (1999) 83:21–37. 10.1016/S0163-7258(99)00010-8 10501593

[10] Druyan S Cahaner A Ashwell CM. The expression patterns of hypoxia-inducing factor subunit alpha-1, heme oxygenase, hypoxia upregulated protein 1, and cardiac troponin T during development of the chicken heart. Poult Sci. (2007) 86:2384–9. 10.3382/ps.2007-00152 17954589

[11] Li M Zhao CJ. Study on Tibetan Chicken embryonic adaptability to chronic hypoxia by revealing differential gene expression in heart tissue. Sci China C Life Sci. (2009) 52:284–95. 10.1007/s11427-009-0005-8 19294354

[12] Bordini M Zappaterra M Soglia F Petracci M Davoli R. Weighted gene co-expression network analysis identifies molecular pathways and hub genes involved in broiler White Striping and Wooden Breast myopathies. Sci Rep. (2021) 11:1776. 10.1038/s41598-021-81303-7 33469097 PMC7815844

[13] Goldstein JA Kelly SM LoPresti PP Heydemann A Earley JU Ferguson EL. SMAD signaling drives heart and muscle dysfunction in a Drosophila model of muscular dystrophy. Hum Mol Genet. (2011) 20:894–904. 10.1093/hmg/ddq528 21138941 PMC3033181

[14] Riquelme P Amodio G Macedo C Moreau A Obermajer N Brochhausen C. DHRs9 is a stable marker of human regulatory macrophages. Transplantation. (2017) 101:2731–38. 10.1097/TP.0000000000001814 28594751 PMC6319563

[15] Ahmed MM Singh P Sultan A Dohare R Tazyeen S Alam A. Unravelling the role of hub genes associated with cardio renal syndrome through an integrated bioinformatics approach. Gene Rep. (2021) 25:101382. 10.1016/j.genrep.2021.101382

[16] Deniz GC Durdu S Dogan Y Erdemli E Ozdag H Akar AR. Molecular signatures of human chronic atrial fibrillation in primary mitral regurgitation. Cardiovasc Ther. (2021) 2021:5516185. 10.1155/2021/5516185 34737791 PMC8538404

[17] Liu DN Zhou YF Peng AF Long XH Chen XY Liu ZL. Helq acts in parallel to Fancc to suppress replication-associated genome instability. Oncol Rep. (2017) 37:1107–113. 10.1093/nar/gkt676 28000895

[18] Anand R Buechelmaier E Belan O Newton M Vancevska A Kaczmarczyk A. HELQ is a dual-function DSB repair enzyme modulated by RPA and RAD51. Nature. (2022) 601:268–73. 10.1038/s41586-021-04261-0 34937945 PMC8755542

[19] Finsterbusch F Ravindranathan R Dereli I Stanzione M Trankner D Toth A. Alignment of homologous chromosomes and effective repair of programmed dna double-strand breaks during mouse meiosis require the minichromosome maintenance domain containing 2 (MCMDC2) protein. PloS Genet. (2016) 12:e1006393. 10.1371/journal.pgen.1006393 27760146 PMC5070785

[20] McNairn AJ Rinaldi VD Schimenti JC. Repair of meiotic dna breaks and homolog pairing in mouse meiosis requires a minichromosome maintenance (MCM) paralog. Genetics. (2017) 205:529–37. 10.1534/genetics.116.196808 27986806 PMC5289834

[21] Alomer RM da Silva EML Chen JR Piekarz KM McDonald K Sansam CG. Esco1 and Esco2 regulate distinct cohesin functions during cell cycle progression. Proc Natl Acad Sci USA. (2017) 114:9906–11. 10.1073/pnas.1708291114 28847955 PMC5604028

[22] Wujcicka WI Kacerovsky M Krekora M Kaczmarek P Grzesiak M. Single nucleotide polymorphisms from CSF2, FLT1, TFPI and TLR9 genes are associated with prelabor rupture of membranes. Genes. (2021) 12:1725. 10.3390/genes12111725 34828331 PMC8620696

[23] Fu Q Mo TR Hu XY Fu Y Li J. miR-19a mitigates hypoxia/reoxygenation-induced injury by depressing CCL20 and inactivating MAPK pathway in human embryonic cardiomyocytes. Biotechnol Lett. (2021) 43:393–405. 10.1007/s10529-020-03045-2 33165673

[24] Xi J Li QQ Li BQ Li N. miR-155 inhibition represents a potential valuable regulator in mitigating myocardial hypoxia/reoxygenation injury through targeting BAG5 and MAPK/JNK signaling. Mol Med Rep. (2020) 21:1011–20. 10.3892/mmr.2020.10924 31922242 PMC7003039

[25] Su JW Fang M Tian B Luo J Jin C Wang XJ. Hypoxia induces hypomethylation of the HMGB1 promoter via the MAPK/DNMT1/HMGB1 pathway in cardiac progenitor cells. Acta Bioch Bioph Sin. (2018) 50:1121–30. 10.1093/abbs/gmy118 30307477

[26] Yan J Song K Bai ZZ Ge RL. WY14643 improves left ventricular myocardial mitochondrial and systolic functions in obese rats under chronic persistent hypoxia via the PPAR alpha pathway. Life Sci. (2021) 266:118888. 10.1016/j.lfs.2020.118888 33310031

[27] Horscroft JA O'Brien KA Clark AD Lindsay RT Steel AS Procter NEK. Inorganic nitrate, hypoxia, and the regulation of cardiac mitochondrial respiration-probing the role of PPAR alpha. FASEB J. (2019) 33:7563–77. 10.1096/fj.201900067R 30870003 PMC6529343

[28] Kurtz M Capobianco E Martinez N Roberti SL Arany E Jawerbaum A. PPAR ligands improve impaired metabolic pathways in fetal hearts of diabetic rats. J Mol Endocrinol. (2014) 53:237–46. 10.1530/JME-14-0063 25122159

[29] Karlstaedt A Schiffer W Taegtmeyer H. Actionable metabolic pathways in heart failure and cancer-lessons from cancer cell metabolism. Front Cardiovasc Med. (2018) 5:71. 10.3389/fcvm.2018.00071 29971237 PMC6018530

[30] Chaudhry A Carthan KA Kang BY Calvert J Sutliff RL Hart CM. PPAR gamma attenuates hypoxia-induced hypertrophic transcriptional pathways in the heart. Pulm Circ. (2017) 7:98–107. 10.1086/689749 28680569 PMC5448534

[31] Liu TJ Yeh YC Lee WL Wang LC Lee HW Shiu MT. Insulin ameliorates hypoxia-induced autophagy, endoplasmic reticular stress and apoptosis of myocardial cells: *in vitro* and *ex vivo* models. Eur J Pharmacol. (2020) 880:173125. 10.1016/j.ejphar.2020.173125 32360347

[32] Saiki C Seki N Furuya H Matsumoto S. The acute effects of insulin on the cardiorespiratory responses to hypoxia in streptozotocin-induced diabetic rats. Acta Physiol Scand. (2005) 183:107–15. 10.1111/j.1365-201X.2004.01375.x 15654924

[33] Liu RF Printz RL Jenkins EC O'Brien TP Te JA Shiota M. Genome-wide gene expression changes associated with exposure of rat liver, heart, and kidney cells to endosulfan. Toxicol In Vitro. (2018) 48:244–54. 10.1016/j.tiv.2018.01.022 29391264

[34] Qi XB Zhang Q He YX Yang LX Zhang XM Shi P. The transcriptomic landscape of yaks reveals molecular pathways for high altitude adaptation. Genome Biol Evol. (2019) 11:72–85. 10.1093/gbe/evy264 30517636 PMC6320679

[35] San JS Du YT Wu GF Xu RF Yang JC Hu JM. Transcriptome analysis identifies signaling pathways related to meat quality in broiler chickens - the extracellular matrix (ECM) receptor interaction signaling pathway. Poultry Sci. (2021) 100:101135. 10.1016/j.psj.2021.101135 33940279 PMC8105667

[36] Fu Q Chen XW Xiang YK. Compartmentalization of beta-adrenergic signals in cardiomyocytes. Trends Cardiovas Med. (2013) 23:250–56. 10.1016/j.tcm.2013.02.001 23528751 PMC4264830

[37] Chen JJ Marsden AN Scott CA Akimzhanov AM Boehning D. DHHC5 mediates beta-adrenergic signaling in cardiomyocytes by targeting G alpha proteins. Biophys J. (2020) 118:826–35. 10.1016/j.bpj.2019.08.018 31547976 PMC7036738

