# Supplementary material for: Effects of chronic hypoxia on the gene expression profile in the embryonic heart in three Chinese indigenous chicken breeds (Gallus gallus)
Source: Front Vet Sci. 2022 Aug 5;9:942159. doi: 10.3389/fvets.2022.942159 (PMC9390884; doi:10.3389/fvets.2022.942159)
Supplement: Supplementary file 1 [file Data_Sheet_1.docx]

Supplemental Table 1. The DEGs in three chicken breeds heart between hypoxia and normoxia

| Chicken breeds | Total | Up-regulated | Down-regulated |
| --- | --- | --- | --- |
| SG | 1050 | 487 | 463 |
| TB | 959 | 508 | 451 |
| DRW | 1060 | 491 | 569 |

Supplemental Table 2. The breed-specific DEGs in three chicken breeds heart between hypoxia and normoxia

| Chicken breeds | Total | Up-regulated | Down-regulated |
| --- | --- | --- | --- |
| SG | 714 | 330 | 384 |
| TB | 653 | 347 | 306 |
| DRW | 712 | 320 | 392 |

Supplemental Table 3. The common DEGs in three chicken breeds heart between hypoxia and normoxia

| Gene symbol | Full name | Functions |
| --- | --- | --- |
| *SGCD* | Sarcoglycan, delta | The protein encoded by *SGCD* is a subcomplex of the dystrophin-glycoprotein complex (DGC). DGC forms a link between the F-actin cytoskeleton and the extracellular matrix. This protein is expressed most abundantly in skeletal and cardiac muscle. Mutations in *SGCD* is associated with autosomal recessive limb-girdle muscular dystrophy and dilated cardiomyopathy. |
| *BIRC7* | Baculoviral IAP repeat containing 7 | *BIRC7* encodes a member of the inhibitor of apoptosis protein (IAP) family, and contains a single copy of a baculovirus IAP repeat (BIR) as well as a RING-type zinc finger domain. The BIR domain is essential for inhibitory activity and interacts with caspases, while the RING finger domain sometimes enhances antiapoptotic activity but does not inhibit apoptosis alone. |
| *HAVCR1* | Hepatitis A virus cellular receptor 1 precursor | *HAVCR1* may be involved in the moderation of asthma and allergic diseases. *HAVCR1* retains a MTTVP amino acid segment that confers protection against atopy. |
| *DHRS9* | Dehydrogenase/reductase (SDR family) member 9 | *DHRS9* demonstrates oxidoreductase activity toward hydroxysteroids and is able to convert 3-alpha-tetrahydroprogesterone to dihydroxyprogesterone and 3-alpha-androstanediol to dihydroxyprogesterone in the cytoplasm, and may additionally function as a transcriptional repressor in the nucleus. |
| *HELQ* | Helicase, POLQ-like | *HELQ* is a single-stranded DNA-dependent ATPase and DNA helicase |
| *DMRT1* | Doublesex- and mab-3-related transcription factor 1 | DMRT1, a transcription factor, is a transcription repressor. |
| *SVOPL* | SVOP-like | *SVOPL* is thought to be a member of solute carrier family 22, which includes transmembrane proteins that transport toxins and drugs from the body. |
| *DZANK1* | Double zinc ribbon and ankyrin repeat domains 1 | *DZANK1* contains two ankyrin repeat-encoding regions. Ankyrin repeats occur in a large number of functionally diverse proteins, mainly from eukaryotes, and are known to function as protein-protein interaction domains. |
| *LOC768686* | Similar to G2 | - |
| *LUZP2* | Leucine zipper protein 2 | This gene encodes a leucine zipper protein. This protein is deleted in some patients with Wilms tumor-Aniridia-Genitourinary anomalies-mental Retardation (WAGR) syndrome. |
| *MCMDC2* | Minichromosome maintenance domain-containing protein 2 | *MCMDC2* may be have ATP binding activity and DNA binding activity and be involved in double-strand break repair. It is predicted to act upstream of or within gamete generation and meiosis I cell cycle process. |
| *ESCO2* | Establishment of sister chromatid cohesion N-acetyltransferase 2 | *ESCO2* may have acetyltransferase activity and may be required for the establishment of sister chromatid cohesion during the S phase of mitosis. |
| *KIAA1217* | KIAA1217 | *KIAA1217* may be involved in embryonic skeletal system development. It may be active in cytoplasm. |
| *CSF2* | Colony stimulating factor 2 | *CSF2* controls the production, differentiation, and function of granulocytes and macrophages. The active form of CSF2 protein is found extracellularly as a homodimer. *CSF2* plays a role in promoting tissue inflammation. |
| *MBOAT1* | Membrane bound O-acyltransferase domain containing 1 | The transmembrane protein encoded by *MBOAT1* is an enzyme that transfers organic compounds, preferably from oleoyl-CoA, to hydroxyl groups of protein targets in membranes. |
| *INADL* | InaD-like | - |
| *CNTNAP5* | Contactin-associated protein-like 5 | *CNTNAP5* may play a role in the correct development and proper functioning of the peripheral and central nervous system and be involved in cell adhesion and intercellular communication. *CNTNAP5* product belongs to the neurexin family, members of which function in the vertebrate nervous system as cell adhesion molecules and receptors. |
| *HNF4A* | hepatocyte nuclear factor 4, alpha | *HNF4A* protein is a nuclear transcription factor which binds DNA as a homodimer. The encoded protein controls the expression of several genes, including hepatocyte nuclear factor 1 alpha. |
| *LOC776590* | Ig kappa chain V-VI region NQ2-6.1-like | - |
| *LTBP1* | Latent transforming growth factor beta binding protein 1 | The protein encoded by *LTBP1* is the family of latent TGF-beta binding proteins. The product of this *LTBP1* targets latent complexes of transforming growth factor beta to the extracellular matrix. |
| *GBP* | Guanylate-binding protein | *GBP* can include interferon-induced proteins that can bind to guanine nucleotides. The encoded protein is a GTPase which hydrolyzes GTP, predominantly to GDP. |
| *BHLHE23* | basic helix-loop-helix family, member e23 | *BHLHE23* contains two highly conserved and functionally distinct domains: the basic domain targets sequence-specific DNA binding, while the helix-loop-helix domain facilitates protein interaction. |
| *NUFIP1* | nuclear fragile X mental retardation protein interacting protein 1 | The protein encoded by *BHLHE23* is associated with the nuclear matrix in perichromatin fibrils and in neurons, and localizes to the cytoplasm in association with endoplasmic reticulum ribosomes. |
| *SGSM3* | Small G protein signaling modulator 3 | *SGSM3* enables GTPase activator and small GTPase binding activity, and is involved in several processes (Rap protein signal transduction; the positive regulation of GTPase activity; and Rab protein signal transduction regulation). |
| *TRIM3* | Tripartite motif containing 3 | This protein encoded by *TRIM3*localizes to cytoplasmic filaments. It is similar to a rat protein which is a specific partner for the tail domain of myosin V, a class of myosins which are involved in the targeted transport of organelles. |
| *SCG2* | Secretogranin-2; Secretogranin II | The protein encoded by *SCG2* is involved in the packaging or sorting of peptide hormones and neuropeptides into secretory vesicles. The full-length protein is cleaved to produce the active peptide secretoneurin. |
| *TBX22* | T-box 22 | *TBX22* encodes the transcription factors involved in the regulation of developmental processes. |
| *ATN1* | Atrophin-1 | The encoded protein includes a serine repeat and a region of alternating acidic and basic amino acids, as well as the variable glutamine repeat. |
| *SLC31A2* | Solute Carrier Family 31 Member 2 | *SLC31A2* may enable copper ion transmembrane transporter activity, and be involved in cellular copper ion homeostasis acts as the upstream of or within regulation of copper ion transmembrane transport. |
| *LOC415913* | high mobility group protein HMGI-C-like | - |
| *ADAMTS3* | ADAM metallopeptidase with thrombospondin type 1 motif, 3 | The preproprotein encoded by *ADAMTS3* is proteolytically processed to generate the mature protease. This protease, a member of the procollagen aminopropeptidase subfamily of proteins, may play a role in the processing of type II fibrillar collagen in articular cartilage. |
| *GRM7* | Glutamate receptor, metabotropic 7 | *GRM7* is linked to the inhibition of the cyclic AMP cascade but differ in their agonist selectivities. |
| *CD109* | CD109 molecule | *CD109* encodes a glycosyl phosphatidylinositol-linked glycoprotein that localizes to the surface of platelets, activated T-cells, and endothelial cells. The protein binds to and negatively regulates signalling by transforming growth factor beta. |
| *SPATA17* | Spermatogenesis associated 17 | *SPATA17* may be located in cytoplasm and have the calmodulin binding activity. |
| *SEC22C* | SEC22 vesicle trafficking protein homolog C | *SEC22C* encodes a member of the SEC22 family of vesicle trafficking proteins. The encoded protein is localized to the endoplasmic reticulum and may play a role in the early stages of ER-Golgi protein trafficking. |
| *SLC9A3* | Sodium/hydrogen exchanger; Solute carrier family 9, subfamily A, member 3 | The protein encoded by this gene is an epithelial brush border Na/H exchanger that uses an inward sodium ion gradient to expel acids from the cell. Defects in this gene are a cause of congenital secretory sodium diarrhea. |
| *PTPRS* | Receptor-type tyrosine-protein phosphatase S | *PTPRS* is known to be signaling molecules that regulate a variety of cellular processes including cell growth, differentiation, mitotic cycle, and oncogenic transformation. |
| *ZNF839* | Zinc finger protein 839 | *ZNF839* may enable metal ion binding activity. |
| *SHOX* | short stature homeobox | *SHOX* belongs to the paired homeobox family and is located in the pseudoautosomal region 1 of X and Y chromosomes. Defects in this gene are associated with idiopathic growth retardation and in the short stature phenotype of Turner syndrome patients. |
| *SLC37A3* | solute carrier family 37, member 3 | *SLC37A3*, the integral component of endoplasmic reticulum membrane, may enable transmembrane transporter activity, and involve in carbohydrate transport and transmembrane transport. |
| *TTR* | Transthyretin isoform 1 precursor | *TTR* is involved in the transport of retinol (vitamin A) in the plasma by associating with retinol-binding protein as well as the intracellular processes including proteolysis, nerve regeneration, autophagy and glucose homeostasis. |
| *PLEKHH1* | Pleckstrin homology domain containing, family H, member 1 | *PLEKHH1* may be located in cytoskeleton. |
| *MYO3B* | Myosin IIIB | *MYO3B* encodes one of the class III myosins. Myosins are ATPases, activated by actin, that move along actin filaments in the cell. |
| *TRPA1* | Transient receptor potential cation channel, subfamily A, member 1 | *TRPA1* protein is highly related to both the protein ankyrin and transmembrane proteins. *TRPA1* may involve a role in signal transduction and growth control. |
| *LOC769232* | - | - |
| *GALNT16* | Polypeptide N-acetylgalactosaminyltransferase 16 | *GALNT16* is predicted to be integral component of membrane and involved in protein O-linked glycosylation via serine and protein O-linked glycosylation via threonine. |
| *ARHGAP32* | Rho GTPase activating protein 32 | *ARHGAP32* related to p75 NTR receptor-mediated signaling, GTPase activator activity, signaling by GPCR, and phosphatidylinositol binding. |
| *SGCD* | Sarcoglycan, delta | The protein encoded by *SGCD* is a subcomplex of the dystrophin-glycoprotein complex (DGC). DGC forms a link between the F-actin cytoskeleton and the extracellular matrix. This protein is expressed most abundantly in skeletal and cardiac muscle. Mutations in *SGCD* is associated with autosomal recessive limb-girdle muscular dystrophy and dilated cardiomyopathy. |
| *DDX43* | DEAD-Box Helicase 43 | The protein encoded by this gene is an ATP-dependent RNA helicase in the DEAD-box family and displays tumor-specific expression. |

Supplemental Table 4. The top 20 up-regulated breed-specific DEGs in SG chicken heart under hypoxia compared to normoxia

| Gene symbol | Log_2_FC | *P* Value | Description |
| --- | --- | --- | --- |
| *LRRTM3* | 4.38 | 3.93E-03 | Leucine rich repeat transmembrane neuronal 3 |
| *UST* | 4.14 | 8.18E-03 | Uronyl 2-sulfotransferase |
| *HNRNPK* | 4.05 | 5.34E-04 | Heterogeneous nuclear ribonucleoprotein K-like |
| *KIAA0430* | 4.03 | 2.62E-03 | KIAA0430 |
| *F13B* | 3.98 | 5.45E-04 | Coagulation factor XIII B chain |
| *LRRFIP1* | 3.68 | 1.38E-02 | Leucine rich repeat (in FLII) interacting protein 1 |
| *BAI3* | 3.59 | 5.34E-03 | Brain-specific angiogenesis inhibitor 3 |
| *CTNNA1* | 3.59 | 1.05E-03 | Catenin (cadherin-associated protein), Alpha 1, 102kDa |
| *DMBX1* | 3.51 | 3.64E-03 | Diencephalon/mesencephalon homeobox 1 |
| *CNNM1* | 3.42 | 1.14E-02 | Cyclin M1 |
| *SCYL3* | 3.39 | 2.68E-03 | SCY1 like pseudokinase 3 |
| *RPL7* | 3.36 | 2.18E-02 | Ribosomal protein L7 |
| *NCOR1* | 3.31 | 3.15E-03 | Nuclear receptor corepressor 1 |
| *MTTP* | 3.25 | 4.17E-04 | Microsomal triglyceride transfer protein |
| *CALML3* | 3.21 | 4.24E-03 | Calmodulin-like 3 |
| *CALN1* | 3.19 | 7.00E-03 | Calneuron 1 |
| *HECW2* | 3.17 | 1.54E-02 | HECT, C2 and WW domain containing E3 ubiquitin protein ligase 2 |
| *TRMT61A* | 3.17 | 4.58E-02 | TRNA methyltransferase 61A |
| *NHLRC3* | 3.15 | 8.48E-04 | NHL repeat containing 3 |
| *LSM14A* | 3.13 | 3.34E-03 | LSM14A mRNA processing body assembly factor |

Supplemental Table 5. The top 20 down-regulated specific DEGs in SG chicken heart under hypoxia

| Gene symbol | Log_2_FC | *P* Value | Description |
| --- | --- | --- | --- |
| EML5 | -4.81 | 2.21E-04 | Echinoderm microtubule associated protein like 5 |
| FAM167A | -4.75 | 5.46E-04 | Family with sequence similarity 167, Member A |
| ATOH7 | -4.63 | 9.80E-03 | Atonal bHLH transcription factor 7 |
| PIK3C3 | -4.46 | 2.22E-03 | Phosphatidylinositol 3-kinase catalytic subunit type 3 |
| CCKAR | -4.45 | 5.21E-03 | Cholecystokinin A receptor |
| KIAA1468 | -4.44 | 1.41E-03 | KIAA1468 |
| CALB1 | -4.26 | 3.87E-02 | Calbindin 1, 28kDa |
| WDR63 | -4.24 | 1.28E-03 | WD repeat domain 63 |
| STARD3NL | -4.16 | 8.79E-04 | STARD3 N-terminal like |
| DOLPP1 | -4.02 | 3.18E-03 | Dolichyldiphosphatase 1 |
| FAM83D | -3.95 | 5.26E-03 | Family with sequence similarity 83, Member D |
| DMBT1L | -3.89 | 1.46E-03 | Deleted in malignant brain tumors 1 protein-like |
| EVC | -3.85 | 1.36E-03 | EvC ciliary complex subunit 1 |
| BRD1 | -3.80 | 5.43E-04 | Bromodomain containing 1 |
| ZFHX4 | -3.79 | 2.50E-04 | Zinc finger homeobox 4 |
| FETUB | -3.71 | 1.79E-02 | Fetuin B |
| RIC8A | -3.61 | 1.80E-03 | RIC8 guanine nucleotide exchange factor A |
| METTL5 | -3.55 | 4.17E-03 | Methyltransferase like 5 |
| LOC771321 | -3.54 | 9.07E-04 | Tyrosine-protein phosphatase non-receptor type 13-like |
| PCDH8 | -3.47 | 1.91E-03 | Protocadherin 8 |

Supplemental Table 6. The top 20 up-regulated specific DEGs in TB chicken heart under hypoxia

| Gene symbol | Log_2_FC | *P* Value | Description |
| --- | --- | --- | --- |
| *DNAJA3* | 4.22 | 2.87E-03 | DNA J heat shock protein family (Hsp40) member A3 |
| *RNF17* | 4.20 | 8.30E-04 | Ring finger protein 17 |
| *NAT10* | 3.99 | 7.44E-04 | N-acetyltransferase 10 |
| *ZNF276* | 3.94 | 7.29E-03 | Zinc finger protein 276 |
| *SDC1* | 3.76 | 3.32E-03 | Syndecan 1 |
| *DNAH9* | 3.61 | 3.26E-03 | Dynein, Axonemal, Heavy chain 9 |
| *SLCO4A1* | 3.60 | 2.33E-04 | Solute carrier organic anion transporter family member 4A1 |
| *RALB* | 3.59 | 2.93E-03 | V-ral simian leukemia viral oncogene homolog B |
| *RNF220* | 3.55 | 3.50E-03 | Ring finger protein 220 |
| *PDLIM5* | 3.53 | 9.50E-04 | PDZ and LIM domain 5 |
| *SAG* | 3.48 | 2.53E-02 | S-antigen; retina and pineal gland (arrestin) |
| *ALDH8A1* | 3.46 | 1.09E-03 | Aldehyde dehydrogenase 8 family member A1 |
| *ENPP2* | 3.41 | 3.51E-03 | Ectonucleotide pyrophosphatase/phosphodiesterase 2 |
| *AGAP1* | 3.37 | 1.79E-03 | ArfGAP with GTPase domain, Ankyrin repeat and PH domain 1 |
| *C9ORF72* | 3.34 | 7.44E-03 | Chromosome Z open reading frame, Human C9orf72 |
| *LOC421486* | 3.31 | 7.00E-03 | Uncharacterized LOC421486 |
| *LOC101748144* | 3.29 | 9.16E-04 | Uncharacterized LOC101748144 |
| *LZTFL1* | 3.22 | 5.71E-03 | Leucine zipper transcription factor like 1 |
| *SLC19A2* | 3.21 | 2.96E-02 | Solute carrier family 19 (thiamine transporter), Member 2 |
| *GZMK* | 3.19 | 9.07E-04 | Granzyme K (granzyme 3; tryptase II) |

Supplemental Table 7. The top 20 down-regulated specific DEGs in TB chicken heart under hypoxia

| Gene symbol | Log_2_FC | *P* Value | Description |
| --- | --- | --- | --- |
| *STK39* | -5.28 | 2.25E-04 | Serine/threonine kinase 39 |
| *ACAD9* | -4.46 | 4.41E-04 | Acyl-CoA dehydrogenase family, Member 9 |
| *RBKS* | -4.12 | 1.96E-02 | Ribokinase |
| *LOC419563* | -4.11 | 2.65E-02 | Interferon-induced guanylate-binding protein 1-like |
| *MPDZ* | -4.09 | 3.21E-03 | Multiple PDZ domain protein |
| *UCP3* | -3.44 | 1.02E-03 | Uncoupling protein 3 (mitochondrial, Proton carrier) |
| *SERPINA1* | -3.44 | 1.90E-02 | Serpin peptidase inhibitor, Clade A, Member 1 |
| *DOCK2* | -3.42 | 4.32E-04 | Dedicator of cytokinesis 2 |
| *DHX57* | -3.42 | 8.95E-03 | DEAH-box helicase 57 |
| *GLIPR1L2* | -3.38 | 2.62E-04 | GLI pathogenesis-related 1 like 2 |
| *LDLRAD1* | -3.35 | 5.93E-03 | Low density lipoprotein receptor class A domain containing 1 |
| *SYNE2* | -3.34 | 9.90E-04 | Spectrin repeat containing, Nuclear envelope 2 |
| *CFAP69* | -3.30 | 3.15E-03 | Cilia and flagella associated protein 69 |
| *CATHB1* | -3.28 | 9.05E-04 | Cathelicidin-B1-like |
| *GCHFR* | -3.26 | 2.21E-02 | GTP cyclohydrolase I feedback regulator |
| *MGAT5B* | -3.17 | 9.39E-03 | Mannosyl-glycoprotein beta-1,6-N-acetyl-glucosaminyltransferase, Isozyme B |
| *KCNJ16* | -3.16 | 5.74E-03 | Potassium voltage-gated channel subfamily J member 16 |
| *ACTRT2* | -3.14 | 9.40E-04 | Actin-related protein T2 |
| *SLC9A5* | -3.12 | 7.63E-04 | Solute carrier family 9, Subfamily A, Member 5 |
| *PANX1* | -3.08 | 1.67E-03 | Pannexin 1 |

Supplemental Table 8. The top 20 up-regulated specific DEGs in DRW chicken heart under hypoxia

| Gene symbol | Log_2_FC | *P* Value | Description |
| --- | --- | --- | --- |
| *AVD* | 5.53 | 5.11E-03 | Avidin |
| *BCL2L15* | 4.06 | 1.63E-04 | BCL2 like 15 |
| *GNL2* | 4.01 | 2.18E-04 | Guanine nucleotide binding protein-like 2 (nucleolar) |
| *SPATS2L* | 3.96 | 2.75E-03 | Spermatogenesis associated, Serine-rich 2-like |
| *LOC427547* | 3.85 | 1.54E-02 | Neural-cadherin-like |
| *ACTL6A* | 3.73 | 7.44E-03 | Actin-like 6A |
| *LOC768776* | 3.68 | 3.08E-04 | Similar to T-cell receptor beta chain |
| *SAMD9L* | 3.56 | 4.30E-02 | Sterile alpha motif domain containing 9-like |
| *RBM22* | 3.43 | 3.69E-02 | RNA binding motif protein 22 |
| *GCNT2* | 3.39 | 8.19E-03 | Glucosaminyl transferase 2, I-branching enzyme |
| *SLCO4C1* | 3.38 | 4.96E-03 | Solute carrier organic anion transporter family, Member 4C1 |
| *LRP11* | 3.32 | 7.62E-04 | LDL receptor related protein 11 |
| *FGFBP2* | 3.24 | 8.05E-03 | Fibroblast growth factor binding protein 2 |
| *C1H11ORF70* | 3.20 | 1.77E-02 | Chromosome 1 open reading frame, Human C11orf70 |
| *GPR141* | 3.18 | 1.09E-02 | G protein-coupled receptor 141 |
| *GSBP9* | 3.17 | 1.19E-02 | Glycosylation site-binding protein |
| *KCNK16* | 3.17 | 1.16E-02 | Potassium channel, Subfamily K, Member 16 |
| *DNAJC6* | 3.16 | 2.25E-02 | DnaJ (Hsp40) homolog, Subfamily C, Member 6 |
| *BNC1* | 3.15 | 8.50E-03 | Basonuclin 1 |
| *TOB2* | 3.14 | 9.22E-04 | Transducer of ERBB2, 2 |

Supplemental Table 9. The top 20 down-regulated specific DEGs in DRW chicken heart under hypoxia

| Gene symbol | Log_2_FC | *P* Value | Description |
| --- | --- | --- | --- |
| *C6* | -4.72 | 9.08E-03 | Complement component 6 |
| *ITPR2* | -4.53 | 2.43E-03 | Inositol 1,4,5-trisphosphate receptor, Type 2 |
| *CCDC18* | -4.49 | 1.14E-02 | Coiled-coil domain containing 18 |
| *SEC63* | -4.35 | 8.41E-04 | SEC63 homolog, Protein translocation regulator |
| *TRPC3* | -4.34 | 8.56E-03 | Transient receptor potential cation channel, Subfamily C, Member 3 |
| *EIF3B* | -4.24 | 1.94E-03 | Eukaryotic translation initiation factor 3 subunit B |
| *RIMKLB* | -4.13 | 3.84E-04 | Ribosomal modification protein rimK-like family member B |
| *JMJD7* | -4.01 | 5.01E-03 | Jumonji domain containing 7 |
| *EXOC4* | -3.87 | 4.08E-03 | Exocyst complex component 4 |
| *COBL* | -3.70 | 4.13E-03 | Cordon-bleu WH2 repeat protein |
| *MMP1* | -3.65 | 2.17E-04 | Matrix metallopeptidase 1 (interstitial collagenase) |
| *LOC421081* | -3.61 | 1.09E-02 | Uncharacterized LOC421081 |
| *PAK2* | -3.53 | 2.88E-03 | P21 protein (Cdc42/Rac)-activated kinase 2 |
| *NFAT5* | -3.49 | 6.13E-03 | Nuclear factor of activated T-cells 5, Tonicity-responsive |
| *RANBP9* | -3.47 | 3.86E-02 | RAN binding protein 9 |
| *GJA8* | -3.47 | 4.10E-04 | Gap junction protein, Alpha 8, 50kDa |
| *ZNF507* | -3.46 | 1.00E-03 | Zinc finger protein 507 |
| *NXT2* | -3.35 | 2.72E-03 | Nuclear transport factor 2-like export factor 2 |
| *DNMT3B* | -3.35 | 1.02E-03 | DNA (cytosine-5-)-methyltransferase 3 beta |
| *FGF13* | -3.30 | 5.02E-03 | Fibroblast growth factor 13 |
